# Pavement Properties and Predictive Durability Analysis of Asphalt Mixtures

**DOI:** 10.3390/polym14040803

**Published:** 2022-02-18

**Authors:** Guohong Zhang, Haonan Wu, Ping Li, Jianhui Qiu, Tengfei Nian

**Affiliations:** 1 Department of Mechanical Engineering, Faculty of Systems Science and Technology, Akita Prefectural University, Akita 015-0055, Japan; wuhaonan199310@163.com (H.W.); qiu@akita-pu.ac.jp (J.Q.); 2 Gansu Provincial Communications, Planning, Survey & Design Institute Co., Ltd., Lanzhou 730030, China; 3 School of Civil Engineering, Lanzhou University of Technology, Lanzhou 730050, China; lzlgliping@126.com (P.L.); tengfeinian@163.com (T.N.)

**Keywords:** durability analysis prediction, pavement performance, dynamic modulus, rutting factor, temperature fatigue factor

## Abstract

The actual lifetimes of many highways are lower than that expected based on the initial pavement design, which brings increasingly prohibitive costs of pavement maintenance and repair. Although many works have been done, the real service lifetimes are still disappointing, and the researchers are also trying their best to increase the projects’ life span. In this study, to comprehensively predict the durability and lifetime of newly designed asphalt mixture structures, an asphalt pavement project consisting of three hot mix asphalt (HMA) mixtures were evaluated. The mixtures were constructed in the pavement project of the Weiwu expressway in Gansu Province. Pavement properties of the asphalt mixtures, rutting and temperature fatigue factors of the dynamic modulus are discussed. The fatigue resistance is supposed to improve on increasing the vehicles’ speed below the freezing point, which may be more suitable for applications in expressways. Meanwhile, the lifetime is measured according to the number of fatigue axle loads calculated, which were corrected between the specimens in the lab and the field core samples. Durability analysis prediction can be obtained based on the fatigue lifetime predictive model accordingly, which can provide more information about the fatigue lifetime and the rehabilitation planning of existing pavements in the future accordingly.

## 1. Introduction

With the ever-increasing capital consumption and environmental burden associated with maintenance activities, sustainable pavement management has recently become a subject of intense discussion [1]. Asphalt mixtures, as visco-elastoplastic materials, are prone to deformations in response to repeated loading into their service period [2]. For many highways, the actual service life has been shorter than that expected from the initial pavement design, as pavement design methods have been moving toward more mechanistic-based approaches [3]. The novel mechanistic-empirical pavement design guide (MEPDG), developed under the National Cooperative Highway Research Program (NCHRP) Project 1-37A, represents a major step in this direction [4]. In these mechanistic-empirical (ME) design procedures, characterization of the mechanical properties of paving materials is essential for determining pavement structures’ responses [5].

Fatigue-related failure is one of the highest forms of asphalt pavement damage [6,7]. Whether a semi-rigid base or a flexible base asphalt pavement, surface cracks appear mainly due to fatigue-related cracking in the long-term perspective [8,9]. Therefore, fatigue design is essential for designing asphalt pavement structures [10,11]. A significant amount of effort has been paid to laboratory assessment and fatigue resistance predictions of asphalt mixtures [12,13,14,15]. Fatigue behavior is typically studied using a phenomenological model that relates failure cycles to the stress/tensile strain. However, this approach only yields information about performance for specific temperature and loading conditions, and it remains very difficult to perform comprehensive evaluations [16]. Zhao [5] reported field measurements and laboratory tests for assessing the short- and long-term effectiveness of hot in-place recycling (HIR). Their results indicate that the HIR with a favorable construction quality is effectual in restoring high-temperature stability and is acceptable for maintaining low-temperature performance and moisture susceptibility. Lin [17] and others performed nondestructive testing of road surface density using an electromagnetic meter, and they developed a quality control/quality assurance (QC/QA) program. The fitting function of experimental QC data and the master curve of a laboratory-derived dynamic modulus were used for evaluating the actual road performance. Joseph [18] and others utilized the dynamic modulus test method for evaluating the road performance of polymer-modified asphalt mixtures derived from corn and soybean oil-derived additives. By obtaining the dynamic modulus master curve, it was shown that corn and soybean bio-derived chemical additives might affect viscous behavior at moderate and higher test temperatures. But the real service lifespan is still disappointed, and the researchers are also trying their best to increase the project’s life span.

## 2. Objectives and Methodology

**Figure 1:**
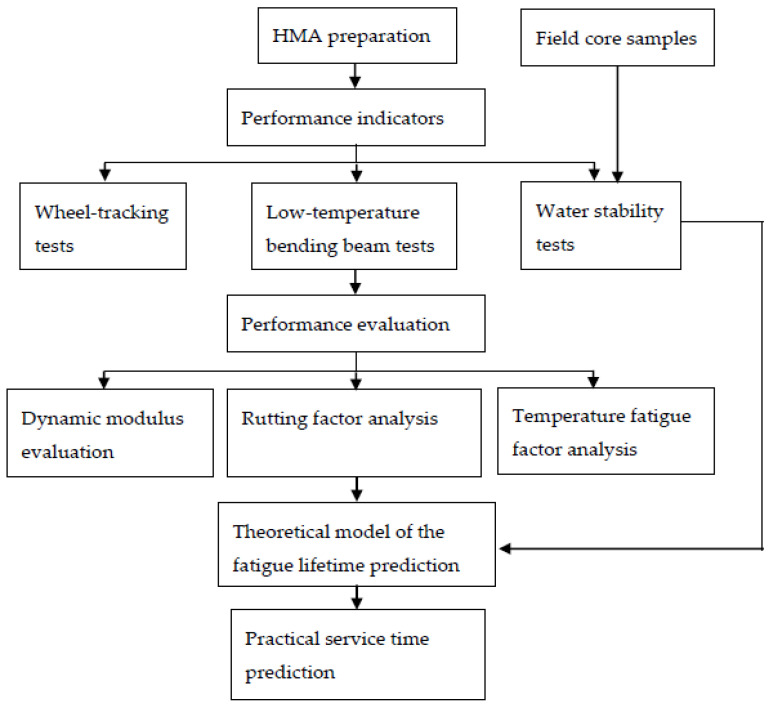
Framework of HMA fatigue lifetime prediction.

**Figure 2:**
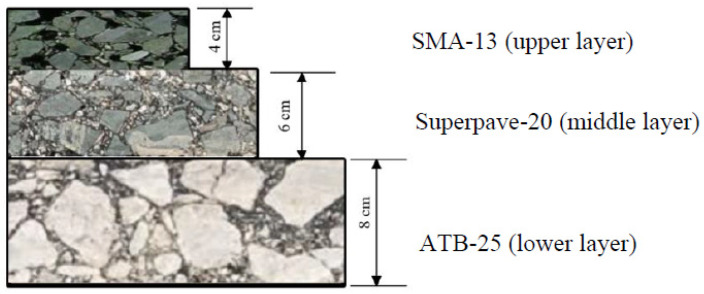
Schematic of the asphalt pavement structure.

An effective analytical framework was developed to comprehensively evaluate the hot-mix asphalt (HMA) mixtures pavement performance and predict the durability and lifetime of newly designed asphalt mixture structures, as presented in Figure 1. Rational scheduling of maintenance or rehabilitation actions in existing asphalt pavements requires systematic knowledge of pavement’s structural integrity during its entire service life [19]. Namely, we considered a stone matrix asphalt (SMA) with a nominal maximum aggregate size (NMAS) of 13 mm (referred to as SMA-13), a supportive mixture with 20-mm-size aggregates (referred to as Superpave-20), and an asphalt-treated base with 25-mm-size aggregates (referred to as ATB-25). Asphalt mixture structures of upper, middle, and lower layers are shown in Figure 2. In this study, pavement properties of the asphalt mixtures, the rutting factors, and temperature fatigue factors from the dynamic modulus have been discussed. Moreover, durability analysis was carried out by comparing and correcting the specimens in the lab and the field core samples. Its practical predictions regarding the lifetime of the Weiwu Expressway project are studied, which is also useful for rehabilitation planning of existing pavements in the future accordingly.

## 3. Experimental Framework

### 3.1. Raw Materials

Two asphalts, including a 70# virgin asphalt (a grade with the needle penetration range of 60–80 mm) meeting the ATB pavement performance grading, and an SBS-modified asphalt with the I-C grade, was used in this research. The 70# virgin asphalt met the grade of PG 70-16, and the specific test results of its properties are listed in Table 1. SBS-modified asphalt was produced by adding 4.5% of the SBS modifier into a 90# virgin asphalt (a grade with the needle penetration range of 80–100 mm). The specific test results are also listed in Table 1, showing that the technical indicators of this asphalt meet the technical requirements of “Technical Specifications for Asphalt Pavement Construction” (JTG F40-2004), “Technical Specifications for Asphalt Pavement Construction (Gansu Provincial Specification)” (DB62/T 3136-2017), and “Implementation and Administration Rules for Asphalt Pavement of Weiwu Expressway Construction Projection (Dingxi Part)”.

### 3.2. Asphalt Mixtures

**Figure 3:**
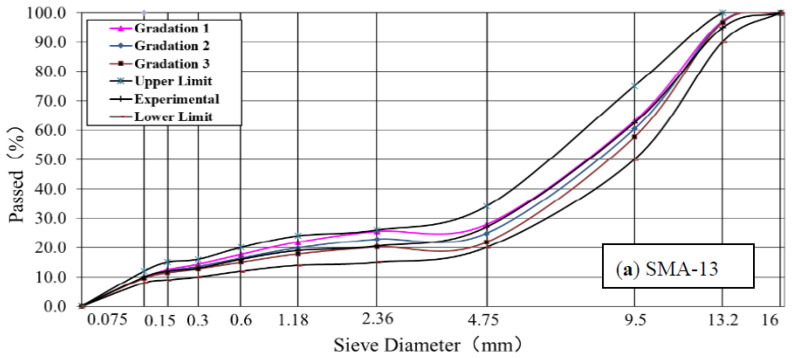

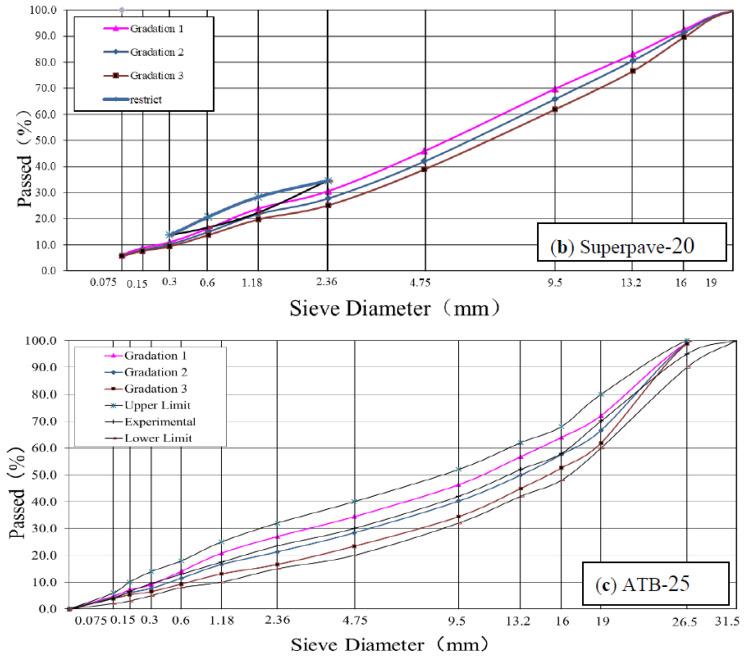
Synthetic gradation curves of the three asphalt mixtures, (**a**) SMA-13, (**b**) Superpave-20, and (**c**) ATB-25.

The mix design process of the asphalt mixtures took the technical specifications of JTG F40-2004 and DB62/T 3136-2017. The Marshall Mix design of ATB-25 and SMA-13 was performed by double-sided compaction of 75 formed Marshall test specimens, mixed with the 70# asphalt and SBS-modified asphalt. The Superpave mix design was performed 100 times to form a test sample and determine the best asphalt content of Superpave-20, which was mixed with the SBS-modified asphalt according to AASHTO T312(TP4). The optimal asphalt content of SMA-13 was 5.9%, such that ATB-25 was 3.8%, and that of Superpave-20 was 4.5%. Synthetic gradations of the three asphalt mixtures are shown in Figure 3, and the volume parameters of the three asphalt mixtures are listed in Table 2. In the table, Gmm is the biggest theory relative density. Pa is an asphalt-aggregate ratio. VMA is the minimum voids of mineral aggregate. VFA is asphalt mixture saturation.

## 4. Measurement Method

### 4.1. Wheel-Tracking Tests

Wheel-tracking tests were carried out according to JTG E20-2011 (T0719). The dimension of the sample was 300 mm (length) × 300 mm (width) × 50 mm (thickness) with the test temperature of 60 °C. The test wheel was a solid rubber tire (outer diameter, 200 mm; wheel width, 50 mm; rubber layer thickness, 15 mm). The running distance of the test wheel was 230 ± 10 mm, and the reciprocating rolling speed was 42 ± 1 cycles/min (21 cycles reciprocating/min). Dynamic stability (DS) was used for evaluating the high-temperature deformation resistance.

### 4.2. Low-Temperature Bending Beam Tests

Low-temperature bending beam tests were carried out according to JTG E20-2011 (T0715). The dimension of the sample was 250 mm (length) × 30 mm (width) × 35 mm (thickness) with the test temperature of −10 ± 0.5 °C and the loading rate of 50 mm/min. Failure strain was used for evaluating the low-temperature crack resistance. The failure strain was significantly correlated with the low-temperature crack resistance.

### 4.3. Freeze-Thaw Splitting Tests

Freeze-thaw splitting tests were conducted according to AASHTO (T283) for Superpave-20 and JTG E20-2011 (T0729) for SMA-13, ATB-25. The tested samples were divided into two groups. The first group was subjected to the unconditioned splitting test, while the other group was subjected to the conditioned test, including vacuum soaking, freezing at −18 ± 2 °C for 16 h, heating in a thermostatic tank at 60 °C for 24 h, and finally immersion into a thermostatic tank at 25 °C for 2 h, before conducting the splitting test. The indirect tension strength ratio (TSR) was used for evaluating water stability.

### 4.4. Dynamic Modulus

**Figure 4:**
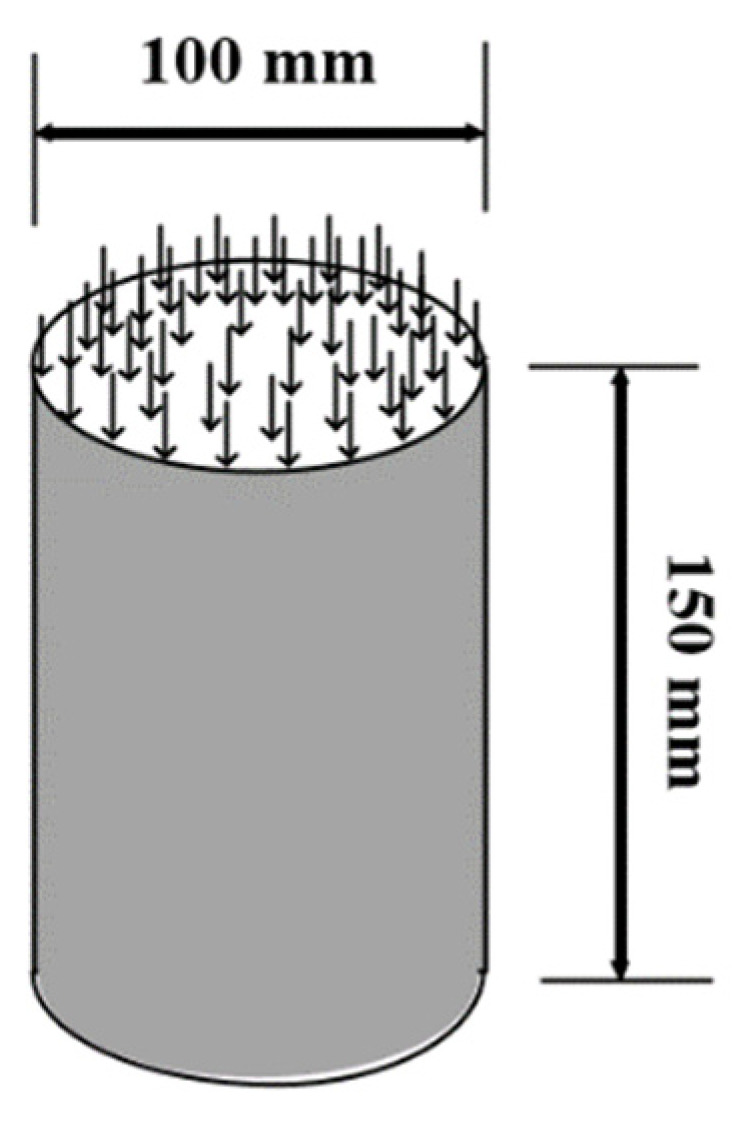
Schematic of the dynamic modulus test sample.

The samples were prepared and compacted by a superpower gyratory compactor (SGC, Troxler, 6015) at an external angle of 1.25° and a vertical pressure of 600 kPa. At first, an asphalt mixture cylinder (diameter, 150 mm; height, 170 mm) was formed using the rotary compactor. After core drilling and sampling, a cylindrical specimen was obtained (diameter of 100 mm and height of 150 mm), where both the upper and lower sides were cut by 10 mm to form the final test specimen (Figure 4). A universal test machine (UTM-100, IPC) to accurately and reliably measure the mechanical response characteristics or parameters of asphalt mixtures was used to measure the dynamic moduli of the studied asphalt mixtures. The test temperatures were −10 °C, 5 °C, 20 °C, 35 °C, and 50 °C, and loading frequencies were 0.5 Hz, 1 Hz, 5 Hz, 10 Hz, 20 Hz, and 25 Hz.

## 5. Results and Discussion

### 5.1. Pavement Performances of Asphalt Mixtures

(1) High-temperature deformation resistance

**Figure 5:**
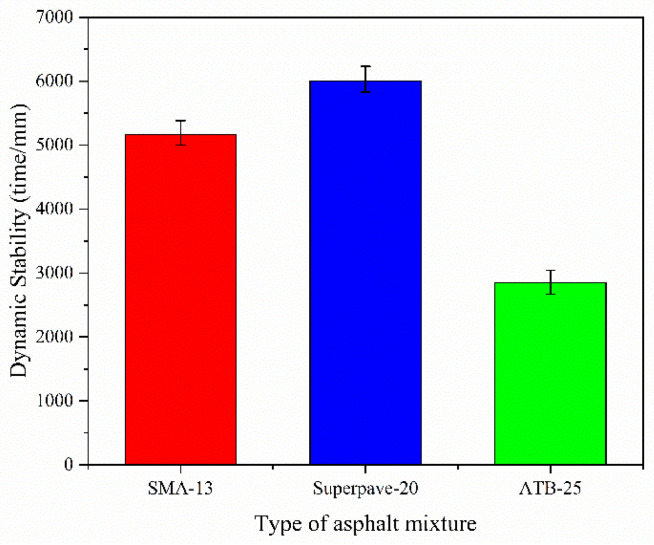
Dynamic stability values for SMA-13, Superpave-20, and ATB-25 asphalt mixtures.

High-temperature deformation resistance was assessed using the wheel-tracking test. According to the results in Figure 5, the DS of the middle layer (Superpave-20) can obtain the highest values, and the lower layer (ATB-25) shows the lowest values. It is suggested that the Superpave-20 pavement shows the best behavior even under a high temperature because the NMAS is large (20 mm). Meanwhile, although the NMAS of SMA is only 13 mm, small than the one of Superpave-20, the DS of SMA-13 can also reach more than 5000 times/mm. It is supposed that the dynamic stability can be improved by the higher content of the asphalt modifier and other additives (such as fibers) in the SMA mixture. By contrast, although the NMAS of ATB is the biggest (25 mm), the DS is less than 3000 time/mm, resulting from the virgin asphalt application rather than the SBS-modified asphalt use. But it can also meet the requirements because ATB-25 constitutes the lowest layer and its temperature does not change significantly compared with the other two pavement layers.

(2) Low-temperature crack resistance

**Figure 6:**
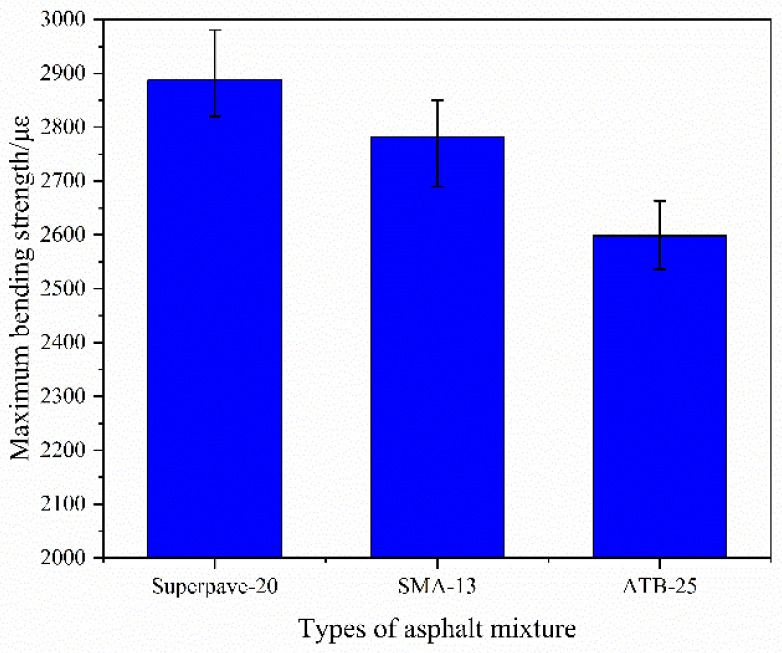
Maximal bending strengths.

Bending beam tests of the asphalt mixtures under low temperature have been conducted, and the maximal flexural strains of the three different structures are shown in Figure 6. Maximal bending strength of Superpave-20, SMA-13, and ATB-25 are 2887.5 µε, 2782.5 µε and 2598.5 µε, respectively. It is suggested that there is excellent crack resistance under low temperature in all the asphalt mixtures. Meanwhile, from the difference of the three maximal bending strength values, Superpave-20 can be more stable and durable with the crack damage attacking under low temperatures.

(3) Water stability performance

In order to evaluate the water stability performances of the asphalt mixtures, water immersion and the freezing-thawing splitting properties were tested. Marshall specimens for SMA-13 and ATB-25 and the cylinder samples for Superpave-20 have been developed according to the DB62/T 3136 and the rotational compaction method. The results are contained in Table 3 and Table 4.

As shown in Table 3, the residual stability values for all the asphalt mixtures are similar to each other. It can be concluded that the three asphalt mixtures showed excellent damage resistance to liquid water. The highest TSR values are shown in Superpave-20, and the lowest ones are shown in ATB-25, according to Table 4. It is proposed that after the freezing and thawing cycles, ATB-25 may be damaged firstly among the three asphalt mixtures.

The pavement performance of the three gradations has been evaluated above. Though all pavement properties have met the design requirements, durability has not been mentioned yet. Therefore, the dynamic modulus tests also have been carried out accordingly.

### 5.2. Rutting Factor Analysis

According to the previous work [20], three isobaric dynamic modulus masters curves of SMA-13, Superpave-20, and ATB-25 have been studied. Pavement road properties of field core samples were all inferiors to the ones of Marshall (ATB-25 and SMA-13), or cylinder (Superpave-20) samples in the lab, and correction factors were derived for evaluating the field durability. From the dynamic modulus, the rutting and temperature fatigue factors can be discussed, which can be used for durability evaluation [21].

The dynamic modulus-tested results from the three gradations under different loading frequencies and temperatures are shown in Table 5. The dynamic modulus values increased as the loading frequency increased under a constant temperature for all of the asphalt mixtures. Meanwhile, with the temperature increasing, the dynamic moduli of all decreased. Moreover, at all temperatures, the dynamic moduli were ATB-25 > Superpave-20 > SMA-13. It can be concluded that the highest dynamic moduli are shown in ATB-25, while the lowest ones are shown in SMA-13. Therefore, with the highest dynamic moduli. ATB-25 is sufficiently suitable for load mitigation when employed as a lower layer. Owing to their lower dynamic modulus, SMA-13 may be quite suitable as the surface layer accordingly.

**Figure 7:**
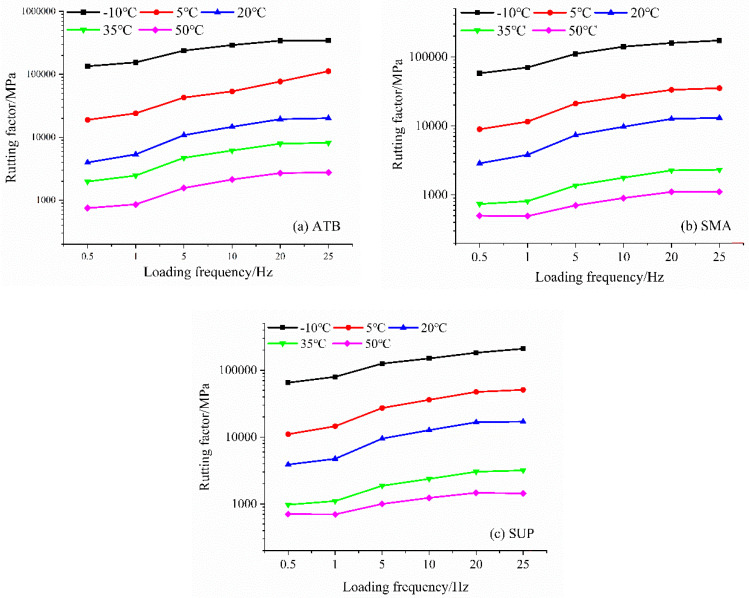
Rutting factors of asphalt mixtures, for different temperatures and loading frequencies. (**a**) is the rutting factors of ATB-25, (**b**) is the ones of SMA-13 and (**c**) is the ones of Superpave-20.

The ratio of the dynamic modulus to the phase angle sine value is proposed in the Strategic Highway Research Program (SHRP) to characterize an asphalt mixture’s ability to resist high-temperature permanent deformations. This value is called the rutting factor and is expressed as indicating a higher dynamic modulus and a smaller phase angle increase the rutting resistance stability of asphalt mixtures under high temperatures. The dynamic modulus can show the mechanical characteristics of the asphalt mixture under different temperatures and loading frequencies; however, it cannot provide accurate information about the rutting resistance of asphalt mixtures under high temperatures. Therefore, the rutting factors of SMA-13, Superpave-20, and ATB-25 have been discussed and shown in Figure 7.

**Figure 8:**
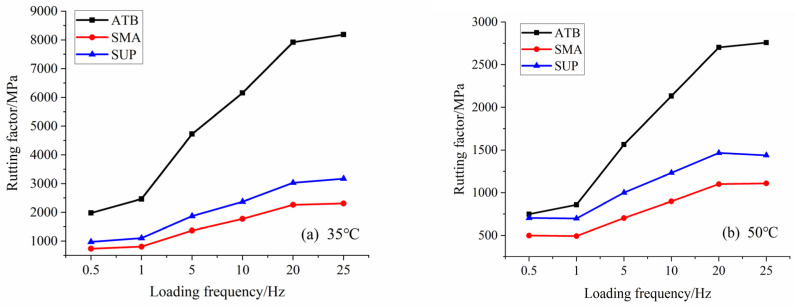
Rutting factors ofasphalt mixtures, for different temperatures and loading frequencies. (**a**) is at 35 °C and (**b**) is at 50 °C.

For all the asphalt mixtures, the rutting factor increased as the loading frequency increased under all of the temperatures. Rutting factors of −10 °C were much higher than the ones of other temperatures. The asphalt stiffness and elasticity at low temperatures are expected, resulting in an excellent rutting resistance performance. Rut usually appears at high temperatures on the road, so the properties under high temperature are much more important. Accordingly, the rutting resistance performances at 35 °C and 50 °C are also shown in Figure 8, in which the rutting factors show the same trends. The rutting factor of ATB-25 shows the largest values, resulting in the best rutting resistance. Although it has met the pavement design requirements, the rutting factors of SMA-13 are the lowest. It is also proposed that SMA-13 may show rut damage over a long time among the three asphalt mixtures.

### 5.3. Temperature Fatigue Factor Analysis

The dynamic modulus and the sine of the phase angle, $\left|E^{*}\right|\sin\delta$, was used for characterizing the fatigue and cracking resistance abilities of the asphalt mixtures under low temperatures. These properties are characterized in terms of the temperature fatigue factor. The lower the temperature fatigue factor, the better the crack resistance. In other words, if the dynamic modulus and the phase angle are low, the asphalt mixture’s ability to resist fatigue and cracking under low temperatures can be improved accordingly.

**Figure 9:**
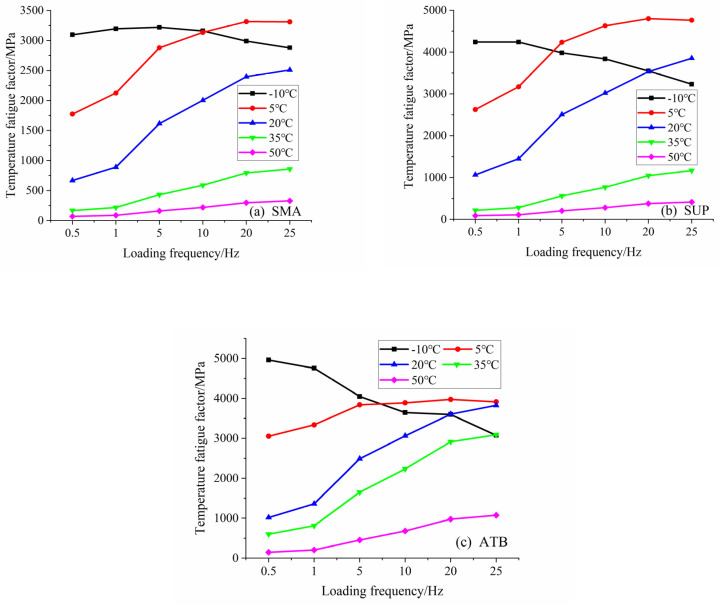
Rutting factors of asphalt mixtures, for different temperatures and loading frequencies. (**a**) is the rutting factors of SMA-13, (**b**) is the ones of Superpave-20 and (**c**) is the ones of ATB-25.

As is shown in Figure 9, when the temperatures are higher than 0 °C (e.g., 50 °C, 35 °C, 20 °C, and 5 °C), the temperature fatigue factors increase with the loading frequency turning faster. It suggests that the lower the speed for temperatures above the freezing point is, the better the fatigue resistance of the pavement turns. The temperature fatigue factor for temperatures under 10 °C decreased with increasing loading frequency. Resulting from the viscoelastic properties of the asphalt, the fatigue resistance increases with increasing vehicle speed for temperatures below the freezing point, which may be more suitable for applications to expressways.

## 6. Predictive Durability Analysis

### 6.1. Theoretical Model of the Fatigue Lifetime Prediction

According to the study above, pavement performance and two factors from the dynamic modulus have been analyzed. But the durability of the project is also paid much attention to. Many factors influence the fatigue time of an asphalt mixture, but mostly by the properties of the asphalt, the contents of the asphalt, porosity, mixing and paving temperature, ambient temperature, and the number of load cycles. Much research has been conducted to determine the factors affecting the fatigue time, which has been studied limiting indoor, such as indoor fatigue tests, full-scale accelerated pavement tests and theoretical studies using finite element methods. In this study, an on-site quality supervision method was employed using modern information technology tools to strictly monitor all of the above parameters during the entire construction process. Therefore, according to the registered values, the equation for predicting the fatigue time was modified by incorporating correction coefficients, which were issued by comparing indoor tests and pavement core drilling sampling tests.

The most widely used method for predicting the fatigue time of an asphalt mixture estimates the number of repeated loads required for a fatigue-related failure [22]. This is captured by a mathematical model that relates the tensile strain at the bottom of the asphalt pavement layer of the asphalt mixture’s dynamic modulus. This model can be defined by the following Equation (1):

(1) $$N_{f}={\mathrm{Ck}}_{1}{\left(\frac{1}{\varepsilon_{t}}\right)}^{k_{2}}{\left(\frac{1}{E}\right)}^{k_{3}}$$

where,

$N_{f}$ is the number of repeated loads during fatigue-related cracking,

$\varepsilon_{t}$ is the tensile strain at the bottom of the asphalt pavement layer,

E is the asphalt mixture’s dynamic modulus,

C is the correction factor; and k_1_, k_2_, and k_3_ are the test regression coefficients.

According to the 2004 AASHTO Design Guide (USA) [22], the equation for predicting the fatigue time is modified based on the Asphalt Institute’s (AI) fatigue model equation. The modified equation is as follows:

(2) $$N_{f}={0.00432k}_{1}^{\prime }C{\left(\frac{1}{\varepsilon_{t}}\right)}^{3.9492}{\left(\frac{1}{E}\right)}^{1.281}$$

where,

C is a function of the asphalt porosity and volume fraction, C = 10^M^,

*E* is the asphalt mixture’s dynamic modulus in the units of psi. Commonly, dynamic moduli obtained in tests are reported in MPa, where 1 psi = 0.006895 MPa,

$\varepsilon_{t}$ is the tensile strain at the bottom of the asphalt pavement layer,

k_1_′ is the correction factor.

The correction factor is obtained using the cracking method, as given by the following Equation (3):

(3) $$M=4.84\left[V_{b}/\left(V_{a}/V_{b}\right)-0.6875\right]$$

where,

*V*_b_ is the asphalt absorption fraction (%),

*V*_a_ is the void fraction (%).

VFA is the effective asphalt saturation of the asphalt mixture and is given by the following Equation (4):

(4) $$\mathrm{VFA}=V_{b}/\left(V_{a}/V_{b}\right)$$

Based on the flexible characteristics of asphalt mixtures rather than cement concrete, the tensile strain at the bottom of the lower layer asphalt pavement is generally used for calculating the fatigue cracking time. In addition, the fatigue cracking model of the constant-strain loading mode is suitable for thin asphalt pavement layers, and the fatigue cracking model of the constant-stress loading mode is suitable for thick asphalt pavement layers [23]. If an asphalt pavement layer has an intermediate thickness (neither thin nor thick), it is a need for a transition relationship between the two above modes.

(5) $$N_{f}=6.32\times 10^{15.96-0.29\beta }k_{a}k_{b}k_{T1}^{-1}{\left(\frac{1}{\varepsilon_{a}}\right)}^{3.97}{\left(\frac{1}{E_{a}}\right)}^{1.58}{\left(\mathrm{VFA}\right)}^{2.72}$$

where,

$N_{f}$ is the fatigue cracking time of the asphalt mixture layer (axis times),

β is the target reliability and the target reliability index (Table 6),

$k_{T1}$ is the temperature adjustment coefficient (where it can be defined as 1.12 in Gansu district, according to JTG D50),

$\varepsilon_{a}$ is the tensile strain at the bottom of the asphalt pavement layer (10^−6^),

k_a_ is the seasonal frozen ground zone adjustment coefficient (Table 7),

$k_{b}$ is the fatigue loading mode coefficient and can be calculated according to Equation (6), as follows:

(6) $$k_{b}={\left[\frac{1+0.3E_{a}^{0.43}{\left(\mathrm{VFA}\right)}^{-0.85}e^{0.024h_{a}-5.41}}{1+e^{0.024h_{a}-5.41}}\right]}^{3.33}$$

where,

$E_{a}$ is the dynamic modulus of the asphalt mixture under 20 °C,

VFA is the asphalt saturation of the asphalt mixture,

$h_{a}$ is the asphalt mixture layer’s thickness (mm).

According to the construction specifics of the Weiwu expressway in this study, the target reliability index β was estimated as 1.65 in the Gansu district, according to JTG D50.

The tensile strain $\varepsilon_{t}$ at the bottom of the asphalt pavement layer can be calculated according to the standard uniaxial and two-wheel load model. The parameters are shown in Table 8.

Therefore, the tensile strain at the bottom of each layer can be calculated according to each layer’s thickness, modulus value, and axle load parameters in the asphalt pavements using the Bisar 2.0 software. The results are shown in Table 9.

As the fatigue-related cracking of an asphalt mixture always starts from its lower layer, the tensile strain at the bottom of the lower layer was used for calculating the fatigue time. A novel fatigue time prediction equation was established and shown below in Equation (7), after incorporating the correction coefficient C of the lower layer calculated above into Equation (5):

(7) $$N_{f}=6.32\times 10^{15.96-0.29\beta }k_{a}k_{b}k_{T1}^{-1}{\left(\frac{1}{\varepsilon_{a}}\right)}^{3.97}{\left(\frac{1}{E_{a}}\right)}^{1.58}{\left(\mathrm{VFA}\right)}^{2.72}C$$

where,

C is the lower correction factor (0.9228).

Eventually, the result of $k_{b}$ = 0.579 can be calculated from Equation (6), and the result $N_{f}$ = 4.58 × 10^9^ (axis order) can also be obtained accordingly.

### 6.2. Practical Service Time Prediction for Weiwu Project

In this study, the design axle load was converted and inferred based on the traffic volume data in the Weiwu expressway design proposal. According to the current road design specifications, according to the initial annual average daily traffic (AADT) volume (two-way) and its vehicle type composition data, the traffic volume of passenger and freight vehicles with two axles and less than four wheels (including four wheels) can be eliminated. Moreover, the ones with 2-axle and 6-wheel data can be derived. The traffic volume for vehicles with more than four wheels (including four wheels, trucks with heavy passenger cars) was used as the initial annual average daily truck traffic (*AADTT*) volume for the two-way design. At the beginning of the two-way direction, the annual average daily traffic volume was multiplied by the direction determination factor (DDF) and the lane determination factor (LDF).

The annual average daily truck traffic volume for the designed lane can be defined using the following Equation (8):

(8) $$Q_{1}=AADTT\times \mathrm{DDF}\times \mathrm{LDF}$$

where,

$Q_{1}$ is the annual average daily truck traffic volume of the designed lane,

AADTT is the annual average daily truck traffic volume for vehicles with at least 2 axles and 6 wheels,

DDF is the direction determination factor (0.5),

LDF is the lane determination factor (set to 0.8 when the number of one-way lanes on the expressway is 2).

The traffic volume is given in the design document, as shown in Table 10:


$$Q_{1}\;=\;2152\;(\mathrm{pcu}/d)$$

According to the available traffic volume growth rate and the design period, the cumulative traffic volume Q of the design lane within the design period can be obtained from Equation (9):

(9) $$Q=\frac{365Q_{1}\left[{\left(1+\gamma \right)}^{t}-1\right]}{\gamma }$$

where,

γ is the traffic volume growth rate;

t is the design period (high-grade highway, 15 years).

The traffic volume growth rate predicted by the project is shown in Table 11.

According to the growth rate in Table 11, the cumulative traffic volume within the design period can be calculated as follows:

(10) $$Q=\frac{365\times 2152\times \left[{\left(1+0.0823\right)}^{15}-1\right]}{0.0823}\;=\;2.17\;\times \;10^{7}(\mathrm{Vehicles})$$

In order to convert the axle load, it is necessary to calculate the truck type distribution coefficient (TTC), which is defined as the ratio of the overall number of trucks and semi-trailers to the overall number of vehicles of various types. According to the existing traffic data, the TTC classification can be determined according to the road TTC classification standard, so the estimated traffic volume in this project is for vehicles of the TTC4 type. Further, the distribution coefficient for vehicles of the TTC4 type can be listed according to the specification shown in Table 12.

Equivalent design axle load conversion factors for various vehicles can be determined according to Equation (11). The ratio of the non-full load vehicle to the fully-loaded vehicle and the equivalent design axle load conversion factor should be obtained, where the values of these two parameters are based on national experience values.

(11) $$\;{EALF}_{m}={EALF}_{\mathrm{ml}}\times {PER}_{\mathrm{ml}}+{EALF}_{\mathrm{mh}}\times {PER}_{\mathrm{mh}}$$

where,

${EALF}_{\mathrm{ml}}$ is the conversion factor of the equivalent design axle load for non-fully loaded vehicles of class m,

${EALF}_{\mathrm{mh}}$ is the conversion factor of the equivalent design axle load for fully loaded vehicles of class m,

${PER}_{\mathrm{ml}}$ is the fraction of non-fully loaded vehicles of class m,

${PER}_{\mathrm{mh}}$ is the fraction of fully loaded vehicles of class m.

Moreover, the daily average equivalent axle number *N*_1_ of the designed lane in the initial year can be calculated according to Equation (12). Similarly, the cumulative action times *N*_e_ of the equivalent design axle load on the design lane can be obtained according to Equation (13).

(12) $$N_{1}=AADTT\times \mathrm{DDF}\times \mathrm{LDF}\times \sum_{m=2}^{11}\left({\mathrm{VCDF}}_{m}\times {EALF}_{m}\right)$$

where,

*AADTT* is the bidirectional annual average daily traffic volume of vehicles with at least 2 axles and 6 wheels (pcu/d),

m is the vehicle type number,

${\mathrm{VCDF}}_{m}$ is the distribution coefficient for vehicles of type m.

(13) $$N_{e}=\frac{\left[{\left(1+\gamma \right)}^{t}-1\right]\times 365}{\gamma }N_{1}$$

where,

$N_{e}$ is the number of equivalent design axle loads on the design lane within the design service life (times);

t is the design service lifetime;

γ is the average annual growth rate of the traffic volume during the design service lifetime.

Accordingly, the calculated results are *N*_1_ = 349,518 (pcu/d) and *N*_e_ = 3.52 × 10^9^ (time), respectively.

Table 13 shows that the number of fatigue axle loads in the design lifetime calculated using the fatigue lifetime prediction equation (*N*_f_ = 4.58 × 10^9^) is greater than the equivalent design axle load times in the design lane in the service lifetime. By contrast, the number of the equivalent design axle load checked against 15-year-long design-related lifetime was *N*_e_ = 3.52 × 10^9^ times. To conclude, the current structure with the lower layer making up the ATB-25 asphalt pavement exhibits a much longer fatigue time. That is to say; the practical fatigue-related cracking time is longer than the design-specified service time for this expressway project.

## 7. Conclusions

In order to predict the durability of newly designed asphalt mixture structures, an asphalt pavement project consisting of three hot mix asphalt (HMA) mixtures was employed. The mixtures were used in the construction of the pavement project of the Weiwu expressway in Gansu Province. Pavement properties of the asphalt mixtures, the rutting factors and temperature fatigue factors from the dynamic modulus are discussed. The fatigue resistance is supposed to improve by increasing the vehicles’ speed below the freezing point, which may be more suitable for applications in expressways.

Meanwhile, the lifetime is measured according to the number of fatigue axle loads calculated, which were corrected between the specimens in the lab and the field core samples. Durability analysis prediction can be obtained based on the fatigue lifetime predictive model accordingly, which can provide more information about the fatigue lifetime and the rehabilitation planning of existing pavements in the future accordingly. Using the novel equation for fatigue lifetime prediction incorporating correction coefficients, the number of fatigue axle loads calculated according to the design lifetime was higher than the equivalent design axle load times in the design lane. This suggests that the practical fatigue-related cracking time is longer than the design service lifespan for the Weiwu expressway project.

According to this study, the real durability and lifetime analysis method was provided, which may be a new way to predict the serving time of the expressway project. Moreover, future rehabilitation planning of existing pavements can also be expected using the novel equation for fatigue lifespan prediction. Finally, these can give helpful advice to designers for new pavement structure design.

## Figures and Tables

**Table 1:** Technical indicators of two asphalt.

Test Item	Test Results (Test Standard: JTG E20-2011)		Test Methods
	70# Asphalt	SBS Modifier Asphalt	
Penetration/0.1 mm (100 g, 5 s), 25 °C	69	75	T0604
Softening point (TR&B)/°C	48.5	84	T0606
Ductility (5 cm/min)/cm	69 (10 °C)	41 (5 °C)	T0605
Mass loss/%	0.03	0.09	T0609
Penetration ratio (25 °C)/%	74	77.3	T0609
Residual ductility/cm	9 (10 °C)	27 (5 °C)	T0604

**Table 2:** Volume parameters of the three asphalt mixtures.

	G_mm_	Pa (%)	VMA (%)	VFA (%)
ATB-25	2.549	3.8	12.8	67.3
Superpave-20	2.684	4.5	13.5	69.6
SMA-13	2.630	5.9	17.0	75.4

**Table 3:** Residual stability values of the three asphalt mixtures.

Residual Stability/%	1	2	3	4	5	6	7	8	Average
SMA-13	81.56	84.41	85.91	88.11	83.47	85.79	84.74	83.08	84.64
Superpave-20	83.07	85.76	84.81	84.66	85.52	85.39	87.86	84.33	85.18
ATB-25	82.62	81.95	86.94	84.42	83.55	86.40	86.08	82.81	84.35

**Table 4:** Freezing-thawing splitting test strength ratio (TSR) of the three asphalt mixtures.

TSR/%	1	2	3	4	5	6	7	8	Average
SMA-13	82.81	87.03	84.97	81.86	82.08	82.99	91.03	88.87	85.21
Superpave-20	89.60	85.63	88.96	87.11	88.32	89.25	86.52	83.01	87.30
ATB-25	78.84	81.72	83.88	76.68	81.11	77.30	83.60	81.88	80.63

**Table 5:** Dynamic modulus test results of the asphalt mixtures.

Mixture Type	Frequency (Hz)	Temperature (°C)									
		−10		5		20		35		50	
		$\left|E^{*}\right|\;(Mpa)$	φ (°)	$\left|E^{*}\right|\;(Mpa)$	φ (°)	$\left|E^{*}\right|\;(Mpa)$	φ (°)	$\left|E^{*}\right|\;(Mpa)$	φ (°)	$\left|E^{*}\right|\;(Mpa)$	φ (°)
ATB-25	0.5	25,884	11.05	8672	26.86	2847	30.41	1245	28.27	424	23.93
	1	28,061	9.76	10,498	24.90	3726	30.28	1595	30.02	523	26.43
	5	32,567	7.14	15,197	19.34	6800	28.70	3060	31.36	987	29.65
	10	34,478	6.07	17,240	17.24	8558	27.20	4091	31.85	1400	30.81
	20	40,325	5.12	19,174	15.16	10,371	25.54	5305	32.21	1881	33.02
	25	40,739	4.33	19,412	14.68	10,519	25.86	5432	33.10	1946	36.70
Superpave-20	0.5	16,634	14.77	5387	29.19	2034	31.57	459	28.12	249	20.68
	1	18,389	13.33	6788	27.84	2617	33.60	557	30.32	276	23.29
	5	22,393	10.24	10,746	23.21	4883	30.92	1025	33.20	453	26.92
	10	24,026	9.19	12,929	20.98	6195	29.19	1349	34.69	588	28.46
	20	25,479	8.01	15,091	18.54	7674	27.41	1781	35.99	746	30.56
	25	26,039	7.13	15,587	17.79	8103	28.40	1925	37.34	772	32.45
SMA-13	0.5	14,275	14.27	4367	29.88	1596	33.86	569	28.48	301	21.95
	1	15,716	12.90	5395	28.65	2059	32.74	673	31.27	322	24.95
	5	19,066	9.93	8352	24.26	3815	31.36	1171	34.16	466	28.49
	10	20,374	8.34	9685	22.26	4863	30.00	1544	35.17	593	29.52
	20	21,720	7.85	11,109	20.30	6010	28.44	2035	36.27	763	31.26
	25	22,128	7.36	11,381	19.85	6251	28.58	2225	37.50	804	32.96

**Table 6:** Target reliability and target reliability indices of different expressway classifications.

Expressway Classification	Expressway	First Class	Second Class	Third Class	Fourth Class
Target reliability (%)	95	90	85	80	70
Target reliability index β	1.65	1.28	1.04	0.84	0.52

**Table 7:** Seasonal frozen ground zone adjustment coefficient k_a_.

Frozen Area	Heavy Cold Area	Intermediate Frozen Area	Light Frozen Area	Other Area
Freezing index F (°C·d) k_a_	2000 0.60–0.70	2000–800 0.70–0.80	800–50 0.80–1.00	≤50 1.00

**Table 8:** Design axle load parameters.

Design Axle Load (kN)	Tire Contact Pressure (MPa)	Single-Wheel Ground Equivalent Circle Diameter (mm)	Center Distance between Two Wheels (cm)
100	0.70	213	319.5

**Table 9:** Tensile strains at the bottom of each layer.

Layer	Upper Layer	Middle Layer	Lower Layer
Bottom tensile strain (με)	18.21	48.87	20.94

**Table 10:** Model and traffic volume table from the design document (2013).

Vehicle Model	Model Number	Daily Traffic Volume (pcu/d)
Passenger car	YU TONG ZK6820G	1436
Motor coach	HUANG HAI DD690	1170
Small truck	YUE JIN NJ131	503
Medium truck	DONG FENG EQ155	1314
Big truck	CHANG ZHENG CZ361	847
Trailer	DONG FENG SP9250	111

**Table 11:** Traffic volume growth rate.

Road Section	2016–2020	2020–2025	2025–2030	2036	Average
Meichuan Interchange—Min County Interchange	9.25%	8.33%	7.67%	7.67%	8.23%

**Table 12:** Distribution coefficient (VCDF_m_) for vehicles of the TTC4 type.

**Vehicle Model (m)**	2	3	4	5	6	7	8	9	10	11
**TTC4**	28.8	43.9	5.5	0.0	9.4	2.0	4.6	3.4	2.3	0.1

**Table 13:** Axle load conversion calculation results.

Axle Load Numbers	$N_{f}\;(\mathrm{Time})$	$N_{e}\;(\mathrm{Time})$
Calculation results	4.58 × 10^9^	3.52 × 10^9^

## Data Availability

Not applicable.
